# Using an Entrustable Professional Activity to Assess Consultation Requests Called on an Internal Medicine Teaching Service

**DOI:** 10.15766/mep_2374-8265.10854

**Published:** 2019-11-22

**Authors:** Angela J. Kang, Katherine Gielissen, Donna Windish

**Affiliations:** 1 Clinical Instructor, Yale Waterbury Internal Medicine Residency Program, Yale University School of Medicine; 2 Clinical Instructor, Department of General Internal Medicine, Yale University School of Medicine; 3 Associate Professor of Medicine, Department of General Internal Medicine, Yale University School of Medicine

**Keywords:** Entrustable Professional Activities, EPAs, Milestones, ACGME, Consults, Intern Competency, Competency-Based Medical Education

## Abstract

**Introduction:**

The Accreditation Council for Graduate Medical Education's milestones require internal medicine residents to have competency in calling consults. Based on a literature review, we developed an Entrustable Professional Activity (EPA) to delineate the knowledge, skills, and attitudes required for a consultation request and, building on the EPA, implemented an assessment instrument to provide feedback to interns calling consultation requests and assess the quality of their consult questions and the level of supervision required in performing this milestone.

**Methods:**

Assessments were done on internal medicine inpatient teaching services. Consultation requests were performed by interns and observed by residents using the assessment instrument. Feedback was provided to the interns. Interns then completed a self-reflection instrument based on the feedback.

**Results:**

Twenty-six paired observations were collected over three 1-month rotations. There was a moderate positive correlation (*r* = .43) comparing resident and intern responses to how they felt about the intern's ability to make a consultation request. There was a strong positive correlation (*r* = .65) comparing resident opinion of how strong the intern's ability in calling a consult to how well the consult question used the PICO (patient, intervention, comparators, outcomes of interest) framework. Twenty-five out of 28 interns (89%) said they would make a change during their next consultation request due to feedback from their resident.

**Discussion:**

Our EPA-based assessment instrument provided an opportunity to give interns feedback and to assess the quality of the consultation requests they made.

## Educational Objectives

By using this assessment, participants will be able to:

1. Directly observe an intern calling a consultation request.
2. Provide structured feedback to the intern about the quality of the consultation request called.
3. Assess the quality of a consult called by an intern using the PICO (patient, intervention, comparators, outcomes of interest) framework and determine how much supervision the intern requires to perform consultation requests moving forward.

## Introduction

The Accreditation Council for Graduate Medical Education's (ACGME's) milestones require internal medicine residents to have competency in calling consultation requests, a regular component of both inpatient and outpatient medicine.^1^ The establishment of milestones such as this one requires the development of valid measures to assess skill attainment.^2^

Calling a consultation request requires the generation of a focused, relevant, clinical question to ask the consultant. To our knowledge, there are no established criteria to measure the effectiveness of a consult question asked; however, an established method for evidence-based research is the PICO (patient, intervention, comparators, outcomes of interest) framework. Its components are ideally suited to assess clinical questions in practice.^3^

Entrustable Professional Activities (EPAs) are units of professional practice that have been proposed as an effective method to assess readiness for independent practice.^4^ EPAs are observable, are measurable, and encompass one or more competencies. EPAs focus on the clinical work to be done and the level of supervision required to perform such work safely and effectively in clinical settings. EPAs are thought to have utility in that they are intuitive to clinical supervisors on the front lines.^5^

The purpose of this assessment was to develop an EPA document with the knowledge, skills, and attitudes required to call a consultation request and then to implement an EPA-based assessment instrument to provide feedback to interns calling consultation requests and assess the quality of the consult question asked and the level of supervision required in performing this milestone.

## Methods

We developed an EPA describing the fundamental knowledge, skills, and attitudes necessary to perform the task of calling a consulting service to obtain subspecialty recommendations on workup and management of a patient's care from the ACGME internal medicine developmental milestones using the framework described by ten Cate et al.^4^

First, we reached consensus on the nature of the task of calling a consultation request. We performed a literature review, revealing no validated measures to evaluate the quality of consultation requests called. One of the more cited articles about consults was by Goldman et al.,^6^ a set of guidelines created in 1983 dubbed “Goldman's 10 commandments,” which were frequently referenced as rules for calling consultation requests. Many of these 10 commandments were rules geared toward the consultant who was being called; however, some applied to the person calling the consultant. The items that were selected to be part of the EPA were the ones that we deemed to be most relevant to an intern in training who would be calling a consultation request and that could be measured using an assessment instrument. The following Goldman principles were incorporated into the EPA document: Determine the question being asked, establish the urgency of the consultation, gather primary data, and communicate as briefly as appropriate.^6^

To assess how well the intern had established the consult question being asked, a literature review was done to determine how best to evaluate the strength of a clinical question. Consult questions were expected to be specific to the patient, with intervention, comparison, and outcome in mind, and geared to a specialist level. The question asked of the consultant was modeled on the PICO framework, as this was both clinically relevant and evidence based.^7^ For example, interns were instructed to state the patient whom the consult was about, a 56-year-old man with new hemoglobin of 6.0 and stable blood pressure; the intervention in question, timing of upper endoscopy; the comparison the consult question was about, waiting until the morning versus emergent endoscopy; and outcome and patient morbidity and mortality. This was the framework around which consult questions were to be asked.

The EPA document was revised through an iterative process involving us and incorporating the narrative review and PICO framework described. Appendix A contains the final EPA document developed, Appendix B contains the Resident Supervisor Instrument, and Appendix C contains the Intern Self-Reflection Instrument. Appendix D notes the items on the Resident Supervisor Instrument that correlate with critical actions and required knowledge, attitudes, and skills on the EPA document. Not all critical actions from the EPA are assessed by items on the Resident Supervisor Instrument. The Resident Supervisor Instrument was developed to assess the critical actions observable while the consultation request was being called. The final critical action on the EPA document, “Intern follows up on consultation recommendations,” was not observable by the resident as the consultation request was being called and was therefore not evaluated on the assessment instrument.

Our assessment was done on two internal medicine inpatient teaching services at Yale New Haven Hospital from April through June of 2018. Participants received approximately 10 minutes of training on the use of the assessment instruments on the first or second day of their inpatient rotation from one of us. The residents and interns were trained either together in the work room or separately in pairs (resident and intern) at a different time if they were unable to be at the group training. Assessment instruments (Appendices B and C) were printed out, and each item was reviewed with the participants along with an explanation: While the intern was calling a consult, the resident was to observe the phone interaction and fill in the Resident Supervisor Instrument. At the end of the call, the resident provided feedback to the intern on the effectiveness of the consultation using the assessment instrument as a tool. If the resident and intern were not able to meet for feedback right after the call, they could do it later that same day. After receiving feedback, the intern completed the Intern Self-Reflection Instrument to identify areas in which improvement was desired based on the feedback he or she had received from the resident. The assessment instruments were reviewed by the residents and interns, and any questions were answered by the author doing the training. The assessment instruments were provided in a folder in the workrooms of the two inpatient teaching services so that they were accessible to the participants to use during the call and after. The completed instruments were stored in a secure folder in the locked resident workroom on each floor and collected three times a week by one of us. While the information was being collected, if the resident teams had any questions about the assessment, they were answered by one of us. The information was disposed of using Health Insurance Portability and Accountability Act secure disposal methods once the data had been deidentified and recorded. None of this information was used as a formal evaluation of the interns or residents. To encourage participation, residents and their corresponding interns received $5 in cash if both completed their assessment instruments. This money was distributed by one of us at the end of the assessment. Please refer to Appendix E for guidelines on how to use the assessment instruments.

The results of the EPA-based assessment activity came from its development in content. The knowledge, skills, and attitudes developed in our EPA were consistent with what is described in the ACGME milestones for competency in calling a consult. The critical actions were based on the available guidelines for calling consults in the literature, including Goldman's 10 commandments.

Results of the intern and resident assessment instruments were aggregated and collected anonymously. A Spearman rank correlation and Pearson correlation were used to compare points on the assessment instrument responses. This work was considered exempt by the Yale University Institutional Review Board.

## Results

Twenty-six paired observations were collected over the course of three 1-month rotations from April through June of 2018. Twenty-six residents and 28 interns (two residents worked with two different interns) responded. Table 1 shows the responses from interns and residents detailing the amount of supervision they felt was required while calling a consult. A moderate positive correlation was found (*r* = .43) comparing question 1 on the Resident Supervisor Instrument (“How do you feel about your intern's ability to call a consult?”) to question 1 on the Intern Self-Reflection Instrument (“How do you feel about your ability to call a consult?”). Furthermore, a strong positive correlation was found (*r* = .65) when comparing the resident opinion of the intern's ability in calling a consult to how well the clinical question asked of the consultant used the PICO framework. Other relevant results from the resident assessment instrument can be found in Table 2.

**Table 1.** Assessment Results Regarding Interns’ Ability to Independently Call a Consult (*N* = 26)

	Unable to		Direct		Indirect		No		Can Supervise	
	Perform Task		Supervision^a^		Supervision^b^		Supervision^c^		Others^d^	
Assessment	No.	%	No.	%	No.	%	No.	%	No.	%
Resident assessment of intern	0	0	1	4	6	23	9	35	9	35
Intern self-assessment	0	0	1	4	2	8	20	77	3	12

a Residents responded to “I would have to make the consultation call as the intern is not ready.” Interns responded to “I would prefer that my senior resident make the consultation call while I observe.”

b Residents responded to “I feel comfortable allowing the intern to make the consult call on their own after they've touched base with me.” Interns responded to “I could make a consultation call after touching base with my senior, knowing they are there if I need them.”

c Residents responded to “The intern would be able to do all the components of a consult call without checking in with me.” Interns responded to “I could make a consultation call without running anything by my senior.”

d Residents responded to “This intern is ready to supervise other residents as they make consultation calls.” Interns responded to “I am ready to supervise residents as they make consultation calls.”

**Table 2.** Supervising Resident (*N* = 26) Assessment Responses While Observing Interns Calling a Consult

Question and Responses	No.	%
How well did the clinical question asked of the consultant use the PICO model?		
The question was not focused.	0	0
The question was focused on 1 but not all the necessary aspects of the PICO model.	0	0
The question was focused on 2 but not all the necessary aspects of the PICO model.	5	19
The question was focused on 3 but not all the necessary aspects of the PICO model.	9	35
The question was focused on all 4 necessary aspects of the PICO model.	11	42
Unanswered	1	4
How clearly did the intern present relevant information about the patient?		
Not enough relevant information was provided.	0	0
Provided some relevant information but not enough to complete consult.	2	8
Concisely provided only relevant information needed for consult.	19	73
Provided mostly relevant but some irrelevant information that was not required for consult.	5	19
Too much irrelevant information was provided.	0	0
Did the intern call within 1 hour of determining the need for consult?		
Yes	20	77
No	6	23
The intern was respectful when calling the consultant.		
Strongly agree	24	92
Somewhat agree	1	4
Neutral	0	0
Somewhat disagree	0	0
Strongly disagree	0	0
Not answered	1	4

Abbreviation: PICO, patient- or problem specific, intervention specific (i.e., prognostic factor, diagnostic test, or treatment), comparison exposure noted (i.e., CT vs. MRI, treatment vs. no treatment), outcome of interest addressed.

A total of 89% of the interns (25 out of 28) stated they would make at least one change when calling their next consult due to receiving structured feedback from their resident. The changes they would make going forward included (1) stating the reason for the consult earlier in the call (25%, seven out of 28), (2) providing more information about the patient to the consultant (21%, six out of 28), (3) creating a more PICO-centered consult question (18%, five out of 28), (4) providing less information about the patient to the consultant (18%, five out of 28), and (5) providing the name and/or medical record number of the patient (7%, two out of 28).

Most interns (90%, 18 out of 20) felt that resident feedback was somewhat or very helpful in improving the way they would subsequently call consults. Six interns left this question blank.

## Discussion

An assessment instrument developed from an EPA about calling a consultation request can be a helpful tool in both providing feedback and assessing the quality of the consult question and the level of supervision required in performing this milestone.

Comparing the supervising resident's assessment instrument to the intern's assessment instrument is a useful way to measure whether a learner's perception of how much supervision he or she needs to call a consultation request aligns with how much supervision a supervisor feels the learner needs. Previous work has shown that the process of direct observation and feedback can enhance resident understanding of intern reliability and subsequently enhance trust.^8^

Furthermore, the assessment instruments we developed may also help in understanding how well an intern is able to self-evaluate his or her milestones. Physicians have limited ability to accurately self-assess their competencies.^9^ This might explain the only-moderate positive correlation found between question 1 on the Resident Supervisor Instrument and question 1 on the Intern Self-Reflection Instrument. The discrepancy between the intern's self-assessment and the external assessment of the supervising resident highlights the importance of outside supervision and evaluation in accurately assessing knowledge, skills, and attitudes. This idea is reinforced by the strong correlation found between the supervising residents’ assessment of the amount of supervision an intern requires and the quality of the PICO question the intern asked. The external assessment by the senior resident had a strong correlation with the more stable and objective measure, the PICO question.

This correlation also provides for an area of further education for calling consultation requests—developing appropriate PICO-centered questions. As calling consults becomes more automated (e.g., placing consult orders into an electronic medical system), elements such as stating the location of the patient and the name of the team calling the consult may become less essential. However, creating appropriate PICO-centered questions will always be important, and thus, this is most likely an important area for further education and resident development.

Although the observations were done by senior residents, they could easily have been done by attendings observing senior residents, consultants observing residents, or interns observing medical students. The assessment instruments lend themselves to a supervisor and learner, not necessarily a resident and intern. This information can be used in clinical competency committees when assessing how well a learner is meeting his or her goals.

The limitations to our assessment include the sample size, which may have affected the strength of the correlation we found. Although, based on our final EPA (Appendix A), we had initially wanted to observe three events per intern, this was not possible due to the timing of the rotations. Therefore, the assessment did not measure improvement in calling consultation requests over time with specific interns. Had three different observations been done per intern, we could have more accurately assessed change in behavior. The EPA document we developed (Appendix A) notes a future goal completion of indirect supervision (level 3) by 6 months into PGY 1. Ideally, this instrument would be used at the beginning of the year to assess interns at the start of their training. The instrument should be tested further, ideally across different institutions with larger sample sizes.

We found that the use of EPA-based assessment instruments is a helpful way to provide feedback and that the instruments require minimal training to use. They are also effective tools to assess the quality of a consult question asked and the level of supervision required by an intern when calling a consultation request on an internal medicine inpatient teaching service.

## Appendices

A. Entrustable Professional Activity.docx
B. Resident Supervisor Instrument.docx
C. Intern Self-Reflection Instrument.docx
D. Resident Supervisor Instrument Correlation EPA.docx
E. Guidelines on How to Use.docx All appendices are peer reviewed as integral parts of the Original Publication.
