# Supplementary material for: Using an Entrustable Professional Activity to Assess Consultation Requests Called on an Internal Medicine Teaching Service
Source: MedEdPORTAL. 2019 Nov 22;15:10854. doi: 10.15766/mep_2374-8265.10854 (PMC6953740; doi:10.15766/mep_2374-8265.10854)
Supplement: Supplementary file 1 — A. Entrustable Professional Activity.docx B. Resident Supervisor Instrument.docx C. Intern Self-Reflection Instrument.docx D. Resident Supervisor Instrument Correlation EPA.docx E. Guidelines on How to Use.docx [file mep-15-10854-s001.zip › A. Entrustable Professional Activity.docx]

**Appendix 1: Final Entrustable Professional Activity**

Title: Call a consulting service to obtain subspecialty recommendations on workup and management of a patient’s care

**Specifications and List of Critical Functions**

Context: Inpatient Internal Medicine service, Verdi 5N of SRC Campus

Target Learner: PGY-1

Limitations: No emergent consultations (ICU, SDU, emergent surgical procedures)

**Critical Actions:**

1) Intern develops appropriate clinical question or reason for consult.

2) Intern prepares or obtains information that would be necessary for consultation (history, labs, imaging, etc).

3) Intern calls consultant and explains the reason for consult and brief history of presentation.

4) Intern effectively answers all clarifying questions.

5) Intern follows up on consultation recommendations.

**Domains of Competence:**

1) Patient Care

2) Interprofessional Skills and Communication

3)Professionalism, Systems

4) Practice Based Learning and Improvement

**Required Knowledge, Attitudes, and Skills:**

1) Identify, classify, and articulate clinical questions as they emerge in patient care activities.

2) Deliver appropriate, succinct, hypothesis-driven presentations.

3) Understand how to prioritize consult calls to ensure prompt completion of clinical tasks.

4) Recognize when to seek additional guidance.

5) Understand how to navigate the various pager services.

6) Effectively communicate with other caregivers and demonstrate professional behavior during interactions.

**Observation Requirement:**

Senior residents (PGY2 and PGY3) or chief residents must do at least three direct observations, including all critical functions, before entrustment decision is made. **Future Goal Completion:**

Indirect supervision (level 3) by 6 months into PGY-1 year.
