# Supplementary material for: Using an Entrustable Professional Activity to Assess Consultation Requests Called on an Internal Medicine Teaching Service
Source: MedEdPORTAL. 2019 Nov 22;15:10854. doi: 10.15766/mep_2374-8265.10854 (PMC6953740; doi:10.15766/mep_2374-8265.10854)
Supplement: Supplementary file 1 — A. Entrustable Professional Activity.docx B. Resident Supervisor Instrument.docx C. Intern Self-Reflection Instrument.docx D. Resident Supervisor Instrument Correlation EPA.docx E. Guidelines on How to Use.docx [file mep-15-10854-s001.zip › B. Resident Supervisor Instrument.docx]

**Appendix B: Resident Supervisor Instrument**

Complete this form while observing your intern. Please CIRCLE your responses.

**1. How do you feel about your intern’s ability to call a consult?**

| I would have to make the consultation call as the intern is not ready. | I would need to be there to help back up the intern as they make the call. | I feel comfortable allowing the intern to make the consult call on their own after they’ve touched base with me. | The intern would be able to do all the components of a consult call without checking in with me. | This intern is ready to supervise other residents as they make consultation calls. |
| --- | --- | --- | --- | --- |

**2. How well did the clinical question asked of the consultant use the PICO model?**

P: Patient or problem specific

I: Intervention specific (i.e. prognostic factor, diagnostic test, or treatment)

C: Comparison exposure noted (i.e. CT vs MRI or treatment vs no treatment)

O: Outcome of interest addressed

| The question was not focused. | The question was focused on 1 but not all the necessary aspects of the PICO model. | The question was focused on 2 but not all the necessary aspects of the PICO model. | The question was focused on 3 but not all the necessary aspects of the PICO model. | The question was focused on all 4 necessary aspects of the PICO model. |
| --- | --- | --- | --- | --- |

­

**3. Was the reason for consult stated at the beginning of the call?**

YES NO

**4. Did the intern use the correct number to contact the consultant on the first try?**

YES NO

**5. How clearly did the intern present relevant information about the patient?**

| Not enough relevant information was provided. | Provided some relevant information but not enough to complete consult. | Concisely provided only relevant information needed for consult. | Provided mostly relevant but some irrelevant information that was not required for consult. | Too much irrelevant information was provided. |
| --- | --- | --- | --- | --- |

**6. Was too much irrelevant information provided to the consultant?**

YES NO

**7. If too much irrelevant information was provided, what was it? CIRCLE all that apply.**

- 1. Too much history (past medical, social, surgical or family)
  2. Irrelevant physical exam findings
  3. Irrelevant lab and/or imaging values
  4. Other (Please specify) ____________________________________

**8. Was too little information provided to the consultant?**

YES NO

**9. If too little information was provided, what wasn’t provided?**

- 1. Not enough history (past medical, social, surgical or family)
  2. Not enough physical exam findings
  3. Not enough lab and/or imaging values
  4. Other (Please specify) ____________________________________

**10. Did the intern provide the patient name or MRN?**

YES NO

**11. Did the intern provide the name of the team calling the consult?**

YES NO

**12. Did the intern call within 1 hour of determining the need for consult?**

YES NO

**13. The intern was respectful when calling the consultant.**

Strongly Somewhat Neutral Somewhat Strongly

Disagree Disagree Agree Agree

**14. Comments about the consult called:**

**15. Comments about the use of this form in providing feedback**
