# Supplementary material for: Using an Entrustable Professional Activity to Assess Consultation Requests Called on an Internal Medicine Teaching Service
Source: MedEdPORTAL. 2019 Nov 22;15:10854. doi: 10.15766/mep_2374-8265.10854 (PMC6953740; doi:10.15766/mep_2374-8265.10854)
Supplement: Supplementary file 1 — A. Entrustable Professional Activity.docx B. Resident Supervisor Instrument.docx C. Intern Self-Reflection Instrument.docx D. Resident Supervisor Instrument Correlation EPA.docx E. Guidelines on How to Use.docx [file mep-15-10854-s001.zip › C. Intern Self-Reflection Instrument.docx]

**Appendix C: Intern Self-Reflection Instrument**

**1. How do you feel about your ability to call a consult? CIRCLE your response.**

| I would prefer that my senior resident make the consultation call while I observe. | I would like my senior to be present while I make the call, to back me up if I need it. | I could make a consultation call after touching base with my senior, knowing they are there if I need them. | I could make a consultation call without running anything by my senior. | I am ready to supervise residents as they make consultation calls. |
| --- | --- | --- | --- | --- |

**2. How helpful was resident feedback in improving the way you will call consults?**

**Very Somewhat Neutral Somewhat Very**

**Unhelpful Unhelpful Helpful Helpful**

**3. Please check off all the changes you expect to make after receiving feedback**

| State the reason for consult earlier |  |
| --- | --- |
| Provide Name and/or MRN of patient |  |
| Create more PICO centered consult questions |  |
| Provide more information about the patient to consultant |  |
| Provide less information about the patient to consultant |  |
| None of the above |  |
| Other (Please write in): |  |

**4. Comments about the feedback you received from your resident:**

**5. Comments about the use of this form:**
