# Supplementary material for: Using an Entrustable Professional Activity to Assess Consultation Requests Called on an Internal Medicine Teaching Service
Source: MedEdPORTAL. 2019 Nov 22;15:10854. doi: 10.15766/mep_2374-8265.10854 (PMC6953740; doi:10.15766/mep_2374-8265.10854)
Supplement: Supplementary file 1 — A. Entrustable Professional Activity.docx B. Resident Supervisor Instrument.docx C. Intern Self-Reflection Instrument.docx D. Resident Supervisor Instrument Correlation EPA.docx E. Guidelines on How to Use.docx [file mep-15-10854-s001.zip › D. Resident Supervisor Instrument Correlation EPA.docx]

Appendix D: Items on Resident Supervisor Instrument that Correlate with Critical Action & Required Knowledge, Attitudes, and Skills on the Final Entrustable Professional Activity

| **Critical Action** | **Items on Resident Supervisor Instrument that address critical action** |
| --- | --- |
| 1) Intern develops appropriate clinical question or reason for consult. | 2 |
| 2) Intern prepares or obtains information that would be necessary for consultation (history, labs, imaging, etc). | 5 |
| 3) Intern calls consultant and explains the reason for consult and brief history of presentation. | 6, 7, 8, 9 |
| 4) Intern effectively answers all clarifying questions. | 7, 9 |
| 5) Intern follows up on consultation recommendations. |  |

| **Required Knowledge, Attitudes, and Skills** | **Items on Resident Supervisor Instrument that address critical action** |
| --- | --- |
| 1) Identify, classify, and articulate clinical questions as they emerge in patient care activities. | 2 |
| 2) Deliver appropriate, succinct, hypothesis-driven presentations. | 3 |
| 3) Understand how to prioritize consult calls to ensure prompt completion of clinical tasks. | 12 |
| 4) Recognize when to seek additional guidance. | 1 |
| 5) Understand how to navigate the various pager services. | 4, 10, 11 |
| 6) Effectively communicate with other caregivers and demonstrate professional behavior during interactions. | 13 |
