# Supplementary material for: Using an Entrustable Professional Activity to Assess Consultation Requests Called on an Internal Medicine Teaching Service
Source: MedEdPORTAL. 2019 Nov 22;15:10854. doi: 10.15766/mep_2374-8265.10854 (PMC6953740; doi:10.15766/mep_2374-8265.10854)
Supplement: Supplementary file 1 — A. Entrustable Professional Activity.docx B. Resident Supervisor Instrument.docx C. Intern Self-Reflection Instrument.docx D. Resident Supervisor Instrument Correlation EPA.docx E. Guidelines on How to Use.docx [file mep-15-10854-s001.zip › E. Guidelines on How to Use.docx]

Appendix E. Guidelines on how to use the Assessment Instruments

Training prior to the start of the rotation should consist of the following:

1. Train residents and interns either together in a work room, or separately by pairs (resident and intern) in the work room at a different time if unable to be at the group training.
2. Review printed assessment instruments (Appendices B and C) and read each item on each assessment.
3. Provide an example of a PICO centered consult question.
4. Explain that while the intern is calling a consult, the resident is to observe the phone interaction and fill in the Resident Supervisor Instrument.
5. Explain that the resident is to then provide feedback to the intern at the end of their call on the effectiveness of the consultation using the assessment instrument as a tool to provide the feedback.
6. Explain how after receiving feedback, the intern is to complete the Intern Self-Reflection Instrument to identify areas where they want to improve based on the feedback he or she received from the resident.

Observation and Feedback Guidelines

1. If they are not able to meet for feedback right after the call, they can do it later that same day.
2. Observations can be done on any consultation called on the phone.
3. Observations can be done when calling any kind of consultant service.
4. There is no limit to the number of observations a pair can do in the day.
5. Either the intern or resident can initiate the observation.

Logistics

1. Assessment instruments should be provided in a folder in the work rooms of the teaching services so they are accessible to the participants to use during and after calling the consultation request.
2. Completed instruments should be stored in a secure folder in the locked resident workroom and collected three times a week by one of the assessment authors.
3. The information should be disposed of using HIPAA secure disposal methods once the data is de-identified and recorded.
4. To encourage participation, residents and their corresponding interns received $5 in cash if both completed their assessment forms, although this is not required to accomplish the assessment.
